# Role of Apolipoproteins and α-Synuclein in Parkinson’s Disease

**DOI:** 10.1007/s12031-017-0942-9

**Published:** 2017-07-10

**Authors:** Fatemeh Nouri Emamzadeh

**Affiliations:** Division of Biomedical and Life Sciences, Faculty of Health and Medicine, University of Lancaster, Lancaster, LA1 4AY UK

**Keywords:** Parkinson’s disease, Apolipoproteins, α-Synuclein

## Abstract

Parkinson’s disease (PD) is a progressive brain disorder that interferes with activities of normal life. The main pathological feature of this disease is the loss of more than 80% of dopamine-producing neurons in the substantia nigra (SN). Dopaminergic neuronal cell death occurs when intraneuronal, insoluble, aggregated proteins start to form Lewy bodies (LBs), the most important component of which is a protein called α-synuclein (α-syn). α-Syn structurally contains hexameric repeats of 11 amino acids, which are characteristic of apolipoproteins and thus α-syn can also be considered an apolipoprotein. Moreover, apolipoproteins seem to be involved in the incidence and development of PD. Some apolipoproteins such as ApoD have a neuroprotective role in the brain. In PD, apoD levels increase in glial cells surrounding dopaminergic cells. However, elevated levels of some other apolipoproteins such as ApaA1 and ApoE are reported as a vulnerability factor of PD. At present, when a clinical diagnosis of PD is made, based on symptoms such as shaking, stiff muscles and slow movement, serious damage has already been done to nerve cells of the SN. The diagnosis of PD in its earlier stages, before this irreversible damage, would be of enormous benefit for future treatment strategies designed to slow or halt the progression of PD. This review presents the roles of apolipoproteins and α-syn in PD and how some of them could potentially be used as biomarkers for PD.

## Parkinson’s Disease

PD is an age-related neurodegenerative disorder characterised by dopaminergic neural cell death in the SN of the brain (Schapira 1995). PD was first described in 1817 by Dr. James Parkinson as ‘the shaking palsy’, and 60 years later was named ‘Parkinson’s disease’ by Jean Martin Charcot, the father of neurology. In PD brains, dopaminergic cell loss in the SN is associated with the formation of cytoplasmic pathological complexes known as Lewy bodies (LBs). LBs can also be found in other parts of the brain, including the hypothalamus, hippocampus and brain stem, but they are more frequent in the SN dopaminergic cells (Duvoisin 1996).

Dopamine loss in PD brains is the reason for motor problems and, possibly, for the cognitive deficits seen in some patients. Motor symptoms of PD include: ‘bradykinesia’, which simply means slowness of movement and is the most common clinical feature of PD; a ‘resting tremor’ that disappears during sleep and voluntary movement; ‘muscular rigidity’ referring to limb resistance to passive movements such as flexion, extension and rotation around a joint; and ‘postural instability’ occurring in the late stage of PD as a result of reduced postural reflexes (Magrinelli et al. 2016; Jankovic 2008). The motor problems usually start with one side of the body and, over time, the disease progresses to involve the other side of the body. A majority of PD patients also experience neuropsychiatric symptoms, mainly depression and anxiety, which can affect the quality of patient life more than motor symptoms (Van Laar and Jain 2004). Other non-motor symptoms of PD can include insomnia, urinary incontinence, sweating, oily skin, impaired olfactory function and dementia and may vary among patients (Salawu et al. 2010). Non-motor symptoms of PD can occur at any stage but mostly they appear before motor symptoms are diagnosed (Van Laar and Jain 2004). The most common feature of PD that is used clinically for detection of the disease is unequivocal bradykinesia. However, PD can also be characterised by the slow ‘pill rolling’ tremor of the patient’s hand when at rest. Moreover, immobile and rigid facial expression and speech slowness are other common characteristics of PD patients (Lees et al. 2009).

α-Syn as the main constituent of LBs (Shults 2006) is shown to be involved in regulation of dopamine levels and so is related to dopamine deficiency and PD. α-Syn interacts with tyrosine hydroxylase (TH) and reduces its activity and consequently decreases dopamine levels (Perez et al. 2002). Moreover, α-syn interaction with protein phosphatase 2A (PP2A) increases the phosphatase activity of PP2A. Activated PP2A dephosphorylates the TH serin 40 residue leading to deactivation of TH and reduction of dopamine levels (Peng et al. 2005; Lou et al. 2010). α-Syn can also bind to the TH gene promotor and downregulate its expression, which consequently results in decreased dopamine levels (Gao et al. 2007). α-Syn is also shown to be involved in regulation of dopamine release from neurons. Overexpression of mutant α-syn can reduce the size of presynaptic dopamine vesicles, interfere with the dopamine exocytosis and increase cytoplasmic dopamine by downregulation of vesicular monoamine transporter 2 (VMAT2) and so leads to elevated oxidation and toxicity of cytoplasmic dopamine (Yavich et al. 2005; Larsen et al. 2006; Lotharius et al. 2002). The pathogenic role of α-syn in dopaminergic neurons may be due to increased production or post-translational modification of the protein, leading to its aggregation. This can occur because of α-syn gene (*SNCA*) mutations, or possibly because of problems in other organelles such as the proteasome system, or mitochondria, or as a consequence of cellular stress (Lesage and Brice 2009; Colzato et al. 2009). In healthy neurons, unwanted α-syn can be cleared by chaperone-mediated autophagy (CMA) and macroautophagy pathways, while mutant α-syn cannot be recognised by CMA, which leads to accumulation of α-syn (Vogiatzi et al. 2008).

## Role of Apolipoproteins in PD

**Fig. 1:**
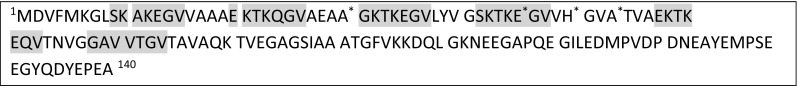
α-Syn protein, which is made of 140 amino acids. Amino acid tandem repeats of [Glu/Gly/Ser]- Lys- Thr- Lys-[Glu/Gln]-[Gly/Gln]- Val-X-X-X-X are highlighted. The star sign shows mutation sites in α-syn including A30P, E46K, H50Q and A53T, which are associated with PD, and all occur in the N-terminal of α-syn in tandem repeats

Apolipoproteins are also reported to be linked to many neurodegenerative disorders. For example, apoE plays a role in many brain disorders, including Alzheimer’s disease, mild cognitive impairment, multiple sclerosis, traumatic brain injury, Creutzfeldt-Jakob disease (CJD) and some others (Tamam et al. 2011; Ponsford et al. 2011; Amouyel et al. 1994.). This raises the possibility that apoE has a significant role in the development of PD. Moreover, studies on the brains of PD transgenic mice show increased cholesterol levels, and because apolipoproteins are involved in the maintenance of cholesterol homeostasis, this seems to suggest a possible link between apolipoproteins and PD (Koob et al. 2010). However, few previous studies have focussed on identification of the role of apolipoproteins in PD. Moreover, apolipoproteins have 11 aa repeats that mediate lipid interactions in a similar way to the N-terminal α-helices of α-syn (Fig. 1). Like α-syn, apolipoproteins, with their amphipathic helices, can be inserted into the membrane and are able to influence its curvature (Varkey et al. 2010). Thus, α-syn and apolipoproteins both have the ability to change membrane structure and to modulate membrane curvature to prevent breakdown of membrane integrity.

Among the apolipoproteins, apoA1 has the greatest similarity to α-syn in its ability to induce membrane curvature (Varkey et al. 2010). In the CNS, apolipoproteins are products of glia cells and neurons take them up for the purpose of axonal growth and synaptic activity. Moreover, apoE is considered to have a neuroprotective role by preventing apoptosis in neurons. The other apolipoproteins such as apoA1 do not have this same protective role in neurons. Among the apoE alleles, apoEε3 seems to have the greatest anti-apoptotic effect. The anti-apoptotic process is initiated when apoE binds to LRP. Moreover, other LRP ligands such as α2 macroglobulins also protect neurons from apoptosis. Thus, apoptosis is regulated by LRP and can be influenced by its ligand apoE. An experiment on rat retinal ganglion cells (RGCs) has shown that when apoE binds to LRP, a calcium signaling pathway is initiated within the RGCs via GSK3β and PKCδ, protecting cells from apoptosis (Hayashi et al. 2007.).

Expression levels of apolipoproteins in the human brain are influenced by the type of apolipoprotein and human age. Among apolipoproteins, the expression levels of apoE, apoD and apoJ are higher in the brain. Moreover, expression levels can change during the different stages of life. For example, apoE levels are 50% higher in the neonatal brain than in an adult person, whereas apoD and apoJ levels are about ten-fold higher in an adult brain than in an embryonic one. Brain cells have specific receptors that bind to a receptor-binding domain of apolipoproteins. These receptor-ligand interactions identify the type of lipoprotein that should be transported to a specific cell. Most of CNS apolipoprotein receptors are family members of the LDL receptor (LDLR), including APOER2, MEGF7, VLDLR, LRP1 and LRP2 (Elliott et al. 2010).

The brain needs unesterified cholesterol mainly for myelin sheaths and in lower amounts in plasma membranes of CNS cells. However, peripheral cholesterol cannot penetrate through the blood-brain barrier (BBB). Therefore, the CNS has its own lipid metabolism pathway. ApoE is the most frequent apolipoprotein in CSF and is secreted by astrocytes. ApoJ, as a small dense apolipoprotein with very low lipid content, is also secreted by astrocytes (Kamino et al. 2002). Therefore, astrocytes are considered the main source of CNS apolipoproteins, although microglia and oligodendrocytes can also produce apolipoproteins. However, some apolipoproteins such as apoAI and apoAII are made peripherally and transported into the CNS via the choroid plexus, in addition to brain endothelial cells, which are able to produce them in lower amounts. Increased cellular cholesterol levels stimulate of expression of lipid transporter proteins on the membrane of astrocytes, microglia and neurons. These ABCA1 and ABCG1 transporters can transfer excess intracellular lipids to apoE and thus control brain lipid equilibrium (Elliott et al. 2010).

## Apolipoprotein E

ApoE is encoded as a 35-kDa protein by a gene containing four exons on chromosomal location 19q13.2. The liver is the biggest generator of plasma apoE, although other organs such as the brain, spleen and kidney can also express apoE (Chen et al. 2001). Similar to other apolipoproteins, apoE is structurally composed of opposing hydrophilic and hydrophobic N-terminal α-helices and a lipid-associated C-terminal domain. The C-terminal domain of apoE initiates an interaction between apoE and the lipid surface of the lipoprotein. The N-terminal domain can then either retain its helix bundle to form a receptor-nonactive conformation or interact with the lipid surface of the lipoprotein and consequently open its helix bundle or interact with the apoE receptor. ApoE can bind to three different receptors including the LDL receptor protein 1, VLDL receptor and apoE receptor 2 (apoER2). Moreover, ApoB has a similar binding region to the apoE LDL-receptor binding region, and both of them, through interaction with the same receptor, control cholesterol levels (Phillips and Apolipoprotein 2014).

ApoE is responsible for the maintenance of cholesterol homeostasis not only in the plasma but also in the CNS. Neuronal cholesterol homeostasis is important for normal neuronal growth, membrane plasticity and synapse development. Therefore, lack of cholesterol in the brain causes impaired neuronal plasticity and reduced neurotransmission, leading to brain damage. In the CNS, apoE from astrocytes is involved in controlling cholesterol levels. The CNS apoE is completely brain-specific, and there is no exchange between plasma-derived apoE and brain apoE because of the boundary of the BBB (Bales 2010).

Genetically, three major alleles are reported for apoE that express three different apoE isoforms. These isoforms are classified based on the amino acids at positions 112 and 158 of the protein. ApoEε2 contains cysteines at both positions, apoEε4 contains arginine at both positions and apoEε3 contains a cysteine at position 112 and an arginine at position 158 (de Knijff et al. 1994; Ghebranious et al. 2005). The WT isoform of apoE in human is considered to be apoEε3 as it is the most frequent form and is least associated with human disorders. However, positions 112 and 158 of apoE in animals contain highly conserved Arg residues, suggesting apoEε4 as the ancestral form of apoE (Raichlen and Alexander 2014).

Characteristically, apoEε4 is not as resistant as apoEε3 to chemical and heat denaturation, while apoEε2 is the most resistant isoform. Moreover, apoEε4 has the highest lipid-binding affinity. These different apoE isoforms are linked to different disorders including neurodegenerative diseases, and apoEε4 has the highest pathological effect among the isoforms (Table 1). For example, expression of human apoE4 in AD neurons also reduces the ratios of SirT1 to SirT2 leading to neurodegeneration (Theendakara et al. 2013). Moreover, those who inherit at least one copy of the ApoE4 allele are more susceptible to developing fragile X-associated tremor/ataxia syndrome (FXTAS) (Silva et al. 2013).

**Table 1:** The role of ApoE in neurodegenerative diseases

ApoE genotype	Effect
ApoEε4/−	ApoE4 binds to Aβ much faster than apoE3 in AD (Strittmatter et al. 1993; Sanan et al. 1994)
	Higher risk of AD in females than males and those without ApoE4 (Payami et al. 1996)
	ApoE4 expression in neuroblastoma cells reduces mitochondrial activity (Chen et al. 2011)
	Increased risk of AD (Corder et al. 1993; Lucotte et al. 1994; Tang et al. 1996; Kawamata et al. 1994)
	Increased neuritic plaques but not fibrillary tangles in AD (Polvikoski et al. 1995)
	Affects the degree of deposition of Aβ in the cerebral cortex of AD females (Wang et al. 2000; Lambert et al. 2001; Pirskanen et al. 2005)
	Is associated with neurofibrillary tangles and senile plaques in AD (Ghebremedhin et al. 2001)
	Faster disease progression in MS patients (Chapman et al. 2001; Liu et al. 2012)
	Decreased hippocampal volume in normal females (Cohen et al. 2001)
	Reduced cognitive ability in normal people (Wilson et al. 2002)
	Shorter survival in males with AD, but not in females (Dal Forno et al. 2002)
	Earlier age of disease onset in PD patients (Zareparsi et al. 2002)
	Decreased hippocampal volume in patients with mild cognitive impairment (Farlow et al. 2004)
	Higher cognitive decline in middle-aged carriers than non-carriers (Blair et al. 2005)
	Declined verbal learning ability in MS patients (Koutsis et al. 2007)
	Is associated with the greater brain atrophy compared to non-carriers in AD patients (Agosta et al. 2009)
	Is related to the lower level of Aβ in the CSF of normal samples than AD patients (Morris et al. 2010)
	Lower Aβ42 levels and higher tau levels compared to non-carriers in AD patients (Kandimalla et al. 2011)
	Increases neurofibrillary tangle and senile plaque counts and decreases brain weight in AD (Wider et al. 2012.)
	Is associated with progressive aphasia in frontotemporal dementia (Acciarri et al. 2006)
ApoEε2/ε2	Longer existence and decreased Alzheimer-type dementia in Down syndrome (Royston et al. 1994)
	Lesser disease severity in MS females (Kantarci et al. 2004)
	Decreased number of neuritic plaques in AD patients (Tiraboschi et al. 2004)
	Is associated with sporadic PD (Huang et al. 2004)
	Decreased risk of AD (Talbot et al. 1994)
ApoEε2/ε3	Earlier age of disease onset in males than females with Huntington diseases (Kehoe et al. 1999)
ApoEε3/ε3	Increased levels of HDL in PD patients (Gregorio et al. 2013)

It seems that apoE is also a risk factor for PD. Some studies have revealed that either increased levels of apoE or elevated levels of its receptor (LRP-1) can lead to PD. The three isoforms of apoE may also play different roles in the pathogenesis of α synucleinopathies (Wilhelmus et al. 2011.). An in vitro study to evaluate the effect of different apoE isoforms on α-syn aggregation shows that apoE4 increases aggregation of α-syn more than other isoforms (Emamzadeh et al. 2016). In another study in Spain, apoEε4 was shown to be more frequent in familial PD, while it was less frequent in sporadic PD when these groups were compared to controls separately. This suggests that apoEε4 may be a risk factor for PD in people with lower genetic variation (Blázquez et al. 2006). However, some other studies show no differences in apoE genotypes (Federoff et al. 2012; Multhammer et al. 2014). In one study, PD patients with dementia (PDD) had two-fold higher apoEε4 allele content than normal people, suggesting a role for apoEε4 in PDD (Parsian et al. 2002). In another study, the apoEε4 allele frequency was found to be higher not only in PDD but also in pure dementia with Lewy bodies (DLB), suggesting a role for apoEε4 in susceptibility to dementia and not PD itself (Tsuang et al. 2013). Other scientists have reported that apoEε4 is a risk factor for earlier age of onset of PD and its frequency is related to susceptibility to PD (Factor et al. 2011). On the whole, the findings suggest that PD patients with at least one apoEε4 allele are more vulnerable to the development of dementia than those who do not have any apoEε4 alleles. Moreover, apoEε4 PD patients have an earlier age of disease onset compared to non-carriers. Therefore, apoEε4 in PD **may be associated with** earlier disease onset and cognitive decline.

## Apolipoprotein J

Clusterin/apolipoprotein J is a 70–80 kDa heterodimeric glycoprotein transcribed from the clu gene at 8p21-p12 and is involved in sperm maturation, lipid transport in association with HDL, membrane remodeling and deactivation of the complement system (Wong et al. 1994). ApoJ consists of two glycosylated subunits with disulfide bonds that connect them together. This apolipoprotein has been found in HDL2, HDL3 and VHDL plasma fractions. ApoJ-HDL particles contain one apoA1 protein molecule for each five apoJ molecules in the HDL structure. Therefore, purified apoJ-HDL from plasma can be identified as different MW bands in Western blotting, including 80, 160, 240, 340 and 520 kDa subclasses (de Silva et al. 1990). ApoJ is involved in reverse cholesterol transport and can efflux excess cholesterol from cells and deliver it back to the liver where cholesterol can be stored or used as a bile acid precursor (Gelissen et al. 1998). The role of apoJ is similar to that of apoA1 and both are involved in the protection of the vascular system against deposition of redundant cholesterol. It seems that apoJ, like apoA1, can cross the BBB although it can also be synthesised in the CNS mainly by astrocytes. It is also assumed to be a chaperone protein that can prevent the aggregation of other proteins. Consistent with this, LBs that contain a high amount of clusterin have less α-syn in their structure, suggesting a protective role for apoJ in preventing α-syn deposition (Sasaki et al. 2002). In AD, increased apoJ levels aid inhibition of Aβ aggregation and accelerate its clearance. Therefore, apoJ polymorphisms may be related to neurodegenerative disorders (Miners et al. 2017). Chaperone activity of clusterin prevents aggregation of α-syn into LBs suggesting a protective role for apoJ in PD (Sasaki et al. 2002).

## Apolipoprotein D

ApoD is mainly expressed in brain, peripheral nerves, placenta, lung, ovary and spleen from a gene at 3p14.2 (Navarro et al. 2013). ApoD’s primary structure is similar to that of lipocalins and constitutes a coiled β-sheet in the form of a barrel that can carry hydrophobic ligands in its central bulk (Rassart et al. 2000). Therefore, this small glycoprotein is not structurally similar to other apolipoproteins. However, as this 27–33-kDa protein can interact with HDL and enter into the plasma membranes, it is considered to have an apolipoprotein activity. These interactions are mediated by apoD hydrophobic residues that lay out on the surface. An elevated level of apoD in the nearby glial cells of SN in PD patients has been reported as well as its increased levels in many other brain injuries related to oxidative damage (Ordoñez et al. 2006). It seems that apoD is an antioxidant cellular protector and that its expression is increased by the interaction of NF-κB to its promoter region, leading to inhibition of lipid peroxidation (Elliott et al. 2010). Especially it has been found that damaged neurons of human brains do not express apoD, while higher expression of apoD in the brainstem seems to be a protective factor against neurodegeneration (Navarro et al. 2013).

## Apolipoprotein A1

ApoA1 is a 28-aa apolipoprotein with ∼28 kD molecular weight and is the main constituent of HDL particles (Brewer et al. 1983). This apolipoprotein is mostly synthesised by the liver and the small intestine and is responsible for gathering extra cholesterol from cells. Structurally, apoA1 contains a bundle of four α-helices at the N-terminal end and two helices at the C-terminal end of the molecule. The C-terminal domain has a high affinity for interaction with lipids and is responsible for the first association of apoA1 with lipoproteins. ApoA1 together with apoE is responsible for lipid transportation and delivery in the brain. However, there is no evidence of apoA1 synthesis in the brain, suggesting that apoA1, in contrast to apoE, can cross the BBB. In AD, apoA1 may be capable of interacting with Aβ and protecting nerve cells from Aβ toxicity. Moreover, in PD, lower levels of one isoform of apoA1 and tetranectin are reported in the CSF of PD patients compared to control individuals, suggesting that apoA1 is a potential biomarker for PD (Wang et al. 2010). It has been recently shown that the plasma level of apoA1 is also a good biomarker for PD as circuit apoA1 is lower in PD patients (Swanson et al. 2015; Qiang et al. 2013). ApoA1 cannot be secreted from neurons, but as the main component of HDL, it is necessary for cholesterol transportation in the brain. Therefore, it possibly passes through the BBB and contributes to the protective roles of HDL. In PD, a lower level of apoA1 means lower efficient HDL and reduced brain cholesterol homeostasis and function (Vitali et al. 2014).

## α-Synuclein

**Fig. 2:**
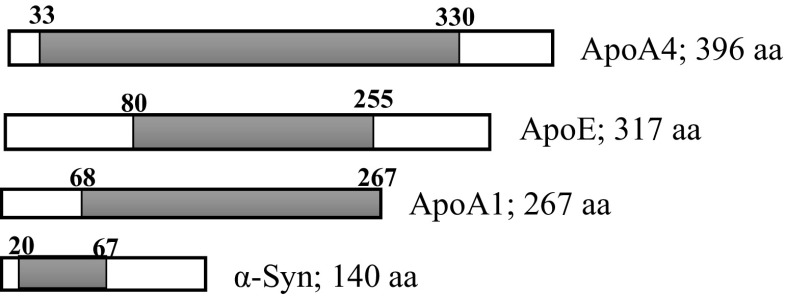
Conserved tandem repeats in apoA4, ApoE, ApoA1 and α-syn. The tandem repeats are located in α-helical regions of apoproteins and α-syn. These repeats induce the helical structure of these proteins and afford them the ability to bind lipids. α-Syn helical structure is important in prevention of β-sheet formation of residues 61–95, which leads to fibrillisation

The term apolipoprotein is usually used to describe either any protein containing lipid components or proteins that can bind to lipids. This lipid binding ability can be based on their hydrophobic structure or through interaction with other lipid-bound proteins. For example, apoD, as a lipid transporter in both CNS and peripheral organs, binds to its ligands through its exposed hydrophobic residues. Therefore, since α-syn can bind to lipids via its highly conserved amphipathic α-helical region, in a way similar to α-helices in apolipoproteins, it can be considered as an apolipoprotein (Emamzadeh 2016) (Fig. 2). Moreover, the detection of α-syn in both CSF and plasma and its role in lipid metabolism all suggest that α-syn could well be a potential member of the apolipoprotein family although it has not been studied or classified as such (Elliott et al. 2010).

Human α-syn is predominantly expressed in the brain in the neocortex, hippocampus, SN, thalamus and cerebellum and is found in LBs. It is encoded by the SNCA, which consists of six exons ranging in size from 42 to 1110 bp (McLean et al. 2000; Yu et al. 2007). Translation of SNCA starts from an ATG start codon that is encoded by exon 2 and stops at a TAA stop codon that is encoded by exon 6. As noted above, the predominant form of α-syn is the full-length protein, but other shorter isoforms have been described. Additionally, C-terminal truncations of α-syn induce aggregation, suggesting that C-terminal modifications might be involved in the pathology of α-syn (Venda et al. 2010). Changes in the levels of α-syn have been reported in CSF and plasma of PD patients compared to control individuals (Hong et al. 2010). Therefore, α-syn can be considered a potential biomarker for PD.

α-Syn aggregates are assumed to be harmful to dopaminergic neurons in the SN and their formation may trigger the transmission of toxic α-syn from affected cells to other adjacent cells, resulting in a cascade of LB formation and, subsequently, cell death. This would promote further dopaminergic cell loss so long as pathogenic α-syn is able to continue to spread to more cells (Luk et al. 2012). This transmission of α-syn between cells could be a normal pathway. However, in a stress situation, the aggregation of α-syn may be initiated within the receiver cells where pre-aggregated α-syn could act as a ‘seed’ to attract more α-syn to aggregate in a ‘prion-like’-like fashion (Alvarez-Erviti et al. 2011; Bernis et al. 2015). Furthermore, because α-syn aggregates should normally be cleared by the proteasome system, or by the lysosomes, any defect in clearance mechanisms could cause the spread of PD pathology as toxic undigested α-syn transmits to other cells. In accordance with this idea, a study has shown that lysosomal inhibition can enhance the amount of insoluble α-syn, leading to the elevated release of exosomes containing toxic α-syn and thus unregulated transmission of pathological α-syn to neighbouring cells, followed by cell death (Luk et al. 2012).

The N-terminal region of α-syn contains five different mutations (A30P, E46K, H50Q, G51D and A53T) that are reported in the familial form of PD. These α-syn N-terminal mutations occur in α-n tandem repeats and affect the conformation of α-syn and lead to α-syn aggregation and fibrillation (Fig. 1). The tandem repeats of α-syn are responsible for the helical secondary structure of α-syn and mutations in this area may trigger β-sheet secondary structure in α-syn and induce fibrillisation (Emamzadeh 2016). In addition, duplication and triplication of the SNCA are also reported in PD (Harada et al. 2009). Dopaminergic cell loss in PD could be a consequence of α-syn aggregation within pathological complexes including LB (Rajagopalan and Andersen 2001). For example, the A30P α-syn mutants have a reduced tendency to interact with membranes and this could lead to α-syn aggregation and the formation of harmful complexes, followed by cell death (Jensen et al. 1998). Moreover, mutant forms of α-syn also damage dopaminergic cells in other ways. WT α-syn plays an antiapoptotic role in neurons via its inhibitory effect on caspase activity. The α-syn A53T mutation destroys this antiapoptotic effect of α-syn and this could lead to cell death in this particular familial form of PD (Alves da Costa et al. 2000).

Moreover, α-syn mutation can accelerate cell death by the effect on the MAPK pathway. α-Syn can interact with mitogen-activated protein kinases (MAPK) through its N-terminal domain, but its C-terminal domain is not involved in this interaction. The α-syn-MAPK interaction is important for regulation of the cell life cycle. α-Syn overexpression is accompanied by reduced active MAPK and increased α-syn-MAPK complexes and this can lead to neuronal death. These findings suggest that the inhibitory effect of α-syn on MAPK can be pathogenic in PD brains where α-syn is overexpressed (Iwata 2001). A mouse model of PD has shown that inhibition of nuclear factor-kappa-light-chain enhancer of activated B cells (NF-κB) as a proapoptotic factor prevents the death of dopaminergic cells (Ghosh et al. 2007). However, a recent study reveals that inhibition of NF-κB cannot save neuroblastoma cells (SH-SY5Y) treated with a toxic agent if α-syn is overexpressed. This means that α-syn inactivates MAPK and triggers apoptosis instead of NF-κB activation. Also, overexpression of A30P α-syn instead of WT α-syn in the same condition reduces the cell damage, suggesting the inefficient inhibitory effect of mutant α-syn on MAPK (Yshii et al. 2015).

Mutant α-syn can also induce mitochondria-dependent apoptosis to a higher extent than the normal protein. Mitochondria are one of the most important organelles in all cell types as they act as ATP machines that provide cells with the required energy content. In the secretory neurons, mitochondria are also involved in the maintenance of Ca2+ levels in a proper amount suitable for vesicle exocytosis and endocytosis in the presynaptic terminals. Therefore, mitochondrial dysfunction is directly linked to reduced cell viability. In dopaminergic neurons, secretion of dopamine can be reduced as a consequence of mitochondrial dysfunction, leading to oxidative damage and cell death through enhanced dopamine metabolism (Ruipérez et al. 2010). The presence of α-syn in the mitochondria from PD brains has been reported, while α-syn is not present in mitochondria isolated from normal brains. Therefore, it seems possible that, in PD, mitochondrial membrane disruption is a consequence of an interaction with α-syn leading to abolished transmembrane potential due to the influx of cations. When mitochondria lose transmembrane potential, ATP production will be reduced because of decreased mitochondrial phosphorylation capacity. Studies show that both WT and mutant α-syn are toxic for mitochondria. However, mutant α-syn aggregates and accumulates in the cytoplasm and cannot and be cleared by proteasomes. Therefore, mutant α-syn has a quicker effect on mitochondrial membrane damage, leading to enhanced neuron death compared to WT α-syn (Moussa et al. 2004). However, although overexpression of α-syn can lead to defective mitochondrial function leading to cell death, α-syn cannot change the mitochondrial morphology. However, MPP+ as an environmental risk factor of PD can induce mitochondrial fragmentation. Interestingly, the effect of MPP+ on mitochondrial segmentation can be treated by the suppression of α-syn expression. Therefore, α-syn is able to alter the mitochondrial morphology in the presence of a risk factor that may induce malfunction of α-syn (Tabrizi et al. 2000). Moreover, poor mitochondrial activity in PD is associated with improper rRNA synthesis and thus impaired ribosomal biogenesis leading to neurodegeneration. Additionally, nucleolin, which regulates rRNA synthesis and ribosomal assembly, can interact with α syn. Therefore, α-syn pathology can affect nucleoli where rRNA is synthesised by affecting nucleolin (Bisaglia et al. 2010).

Additionally, the mutation in α-syn can increase susceptibility to dopamine toxicity. Although WT α-syn is vulnerable to fibril formation and aggregation, mutant α-syn aggregates faster. Moreover, mutant α-syn increases neuronal sensitivity to dopamine toxicity compared to WT α-syn (Conway et al. 2001). The toxicity of dopamine in mutant α-syn neurons can be related to monoamine transporter dysfunction induced by the mutant protein, since the affected monoamine transporter is not capable of compartmentalising dopamine within vesicles, and this leads to oxidative stress. It seems that overexpression of WT α-syn can cause similar toxicity, but over a longer time (Martinez et al. 2003). Moreover, treatment of neuroblastoma cells with dopamine supplement showed increased aggregation of α-syn (Ostrerova-Golts et al. 2000), suggesting that dopamine not only aggregates α-syn but also forms cytosolic quinones that stabilise these structure (Bisaglia et al. 2010). Oxidative species of dopamine in the cytoplasm oligomerise α-syn into harmful aggregates that inhibit the activity of chaperone-mediated autophagy leading to neurodegeneration (Conway et al. 2001).

In addition, mutant α-syn is more vulnerable to aggregation in the presence of metal ions. A study on human neuroblastoma cells has revealed that aggregation of both WT and mutant α-syn in complex with ubiquitin can be induced by iron and ROS. Moreover, the amount of α-syn expression and the type of α-syn are linked to the number of aggregates in the neurons. Mutant α-syn causes more aggregation than WT α-syn. Moreover, the A53T mutation stimulates the formation of more α-syn aggregates than A30P, while the A30P mutation has a higher effect on α-syn aggregation than WT α-syn. Therefore, in PD, cell death could be caused by α-syn aggregation induced by iron and other radical initiators (Martinez et al. 2003).

Inhibition of proteasomal function is another effect of mutant α-syn. The ubiquitin-proteasome pathway detects and degrades aggregated damaging proteins in the cytoplasm. Accumulation of polyubiquitinated proteins activates 26S proteasome and unfolded protein aggregates by its 19 S regulatory complex and cleaves them by its 20S proteolytic particle. In PD accumulation of LBs in dopaminergic cells cannot be cleared out as this pathway is abolished. Mutations in E3 ubiquitin-protein ligase, parkin (Ostrerova-Golts et al. 2000) and ubiquitin C-terminal hydrolase L1 (UCH-L1) (Leroy et al. 1998) have been previously reported in familial PD indicating the role of this mechanism in neuroprotection. It seems that α-syn aggregates can also damage this pathway. In Lewy bodies the 20S proteasome subunit binds to α-syn and inhibits its hydrolytic activity (Lindersson et al. 2004). Both α-syn mutants (A53T and A30P) are related to increased α-syn aggregation in familial PD. Consequently, both ubiquitin-dependent and -independent proteosomal activities of 26S proteasomes are abolished by α-syn aggregates. These data raise the possibility that the toxicity of α-syn depends on its inhibitory effect on proteosomal function due to the formation of proteolysis-resistant α-syn aggregates (Stefanis et al. 2001).

Furthermore, impairment of the actin cytoskeletal structure and dynamics can be induced by A30P mutation. The interaction of α-syn with actin causes downregulation of actin polymerisation and acceleration of its depolymerisation. Therefore, α-syn is involved in the modulation of cytoskeletal dynamics during microfilament reassembly. In contrast, A30P α-syn accelerates polymerisation of actin during cytoskeleton reassembly, which disrupts not only the cytoskeletal dynamics but also other cytoskeletal-related processes. Therefore, the A30P mutation could be involved in the pathogenesis of PD by affecting the cytoskeletal structure (Sousa et al. 2009).

## Possible Pathogenic Effects of α-Syn Transmission

It has been shown that young grafted neurons from aborted embryos start to form LBs a few years after transplantation to PD brains. These results suggest that toxic α-syn can transfer from one part of the brain to another consistent with the Braak hypothesis. Braak’s group believes that in PD some type of brain pathogen moves from the gut to the midbrain and then to the brain cortex and, clearly, α-syn could act as such a pathogen (Braak et al. 2003). These findings raise some interesting questions about how α-syn leaves the transfer cells and enters into other cells, as well as how exogenous α-syn can cause harm to recipient cells. Some scientists suggest that toxic transferred α-syn may become a template that initiates aggregation of unfolded α-syn in the receiver cells. It is also possible that entrance of α-syn in the recipient cells triggers cellular stress, leading to the formation of misfolded and aggregated proteins (Steiner et al. 2011).

α-Syn may leave cells via an exocytosis mechanism. Disequilibrium between unfolded α-syn and aggregated species may both induce self-aggregation of α-syn and sequester other proteins leading to the formation of LBs. In this circumstance, autophagosomal and lysosomal functions may be disturbed causing the dispatch of exosomes contacting α-syn. These exosomes reach other cells and bind to their membranes discharging α-syn to the cytoplasm of recipient cells. Moreover, as dying neurons discharge their protein contents into the extracellular space, free α-syn (without the protection of exosomes) may transfer to recipient cells. This is possible based on the ability of α-syn to interact with membrane lipids (Steiner et al. 2011).

## Possible Pathogenic Effect of α-Syn Truncation

The mutations in α-syn can not only lead to the formation of a toxic protein, but also C-terminal truncation can have a similar effect. C-terminally shortened α-syn (α-synΔC) aggregates much faster than full-length α-syn (α-synFL) and these kinds of aggregate are common in familial forms of PD. An in vitro experiment has proven that aggregation of α-synFL can be induced in the presence of α-synΔC. Moreover, co-expression of α-synFL with α-synΔC in rats can cause neuronal death as the consequence of elevated α-synFL aggregates while the expression of α-synFL by itself in the rat brain just leads to moderate aggregation without any neuronal death (Ulusoy et al. 2012).

The formation of α-synΔC is a normal process in cells that typically leads to accumulation of α-syn, but it is not toxic in the usual circumstance. However, in familial PD, the raised levels of both soluble α-syn and truncated forms are responsible for a greater number of inclusions and amount of cell death. Therefore, the amount of α-synΔC levels in neurons is positively related to the synucleinopathy intensity. The sites of α-syn truncation at the C-terminus may be Asp119/Pro120, Asn122/Glu123 and Glu123/Ala124. It seems that α-syn cleavage is mediated by different proteases and the proteasome is believed to be responsible for the cleavage of α-synFL at the Asp119/Pro120 position, while calpain1 mediates the truncation of α-synFL at the Asn122/Glu123 position (Li et al. 2005).

## Conclusion

In conclusion, apolipoproteins that are involved in cholesterol removal from the cells and transport excess cholesterol to bile seem to have a protective role in PD. HDL particles are the major lipoproteins that remove cholesterol from the arteries and send them back to the liver. Therefore, HDL and ApoA1 as the main constituents of HDL are very important to prevent cardiovascular disease such as atherosclerosis, which is result of accumulation of extra cholesterol in the blood. ApoA1 is also has a protective role in PD. It is consistent with findings that show people who have lower heart rate variability are at a higher risk of PD (Alonso et al. 2015). ApoD is also very important in cholesterol hemostasis as it mediates the interaction between HDL and LDL. In case of nerve damage, apoD levels increase and mediate transportation of HDL particles to the damaged site (Braesch-Andersen et al. 2014.). Therefore elevated levels of apoD in glial cells of SN can be associated with PD. However, apoE is involved in cholesterol transport to the cells and provides the cholesterol for the brain (Puglielli et al. 2003). ApoE despite apoA1 is associated with cardiovascular disease due to formation of cholesterol plaques within the arteries. An elevated level of apoE is also a risk factor for Alzheimer’s disease and possibly can be involved in PD. α-Syn like apolipoproteins also has the ability to interact with cholesterol and amino acids 67–78 mediate this interaction (Fantini et al. 2011). Extracellular α-syn can thus bind to the membrane of neurons and glial cells and internalise into them and propagate in a prion-like fashion. Cholesterol also mediates the interaction of oligomeric α-syn with the cell membrane, which leads to membrane disruption and cell death (van Maarschalkerweerd et al. 2015). Taken together, high levels of cholesterol circulating in body fluids may increase the risk of PD. Therefore, those particles such as apoA1 that decrease cholesterol levels may reduce the PD rate. However, particles that increase cholesterol levels such as apoE may lead to PD.

AD, Alzheimer’s disease; Apo, Apolipoprotein; BBB, Blood-brain barrier; CMA, Chaperone-mediated autophagy; CNS, Central nervous system; CSF, Cerebrospinal fluid; DLB, Dementia with Lewy bodies; HDL, High-density lipoproteins; kDa, Kilodalton; LBs, Lewy bodies; LDL, Low-density lipoproteins; MAPK, Mitogen-activated protein kinases; MAPT, Microtubule-associated protein tau; MW, Molecular weight; PD, Parkinson’s disease; PEA, β-Phenylethylamine; PP2A, Protein phosphatase 2A; ROS, Reactive oxygen species; SN, Substantia nigra; TH, Tyrosine hydroxylase; VLDL, Very-low-density lipoproteins
